# Developing Medical Student Competencies, Clinical Skills, and Self-Efficacy With an Emergency Medical Responder Certification Course

**DOI:** 10.7759/cureus.26678

**Published:** 2022-07-09

**Authors:** Brandon Tapasak, Max McCall, Elliott Cheung, Richard Peppler

**Affiliations:** 1 College of Medicine, University of Central Florida College of Medicine, Orlando, USA; 2 Academic Affairs, University of Central Florida College of Medicine, Orlando, USA

**Keywords:** self-efficacy, competency, gap years, medical education, emergency medical responder

## Abstract

Introduction

Previous studies have claimed gap year clinical experiences before medical school matriculation increase student self-efficacy and clinical confidence. At the University of Central Florida College of Medicine, 41 first-year medical students participated in a new certification course to become emergency medical responders before beginning their coursework. This study describes the results of a follow-up study that aims to investigate the impact the course had on student competency, self-efficacy, and clinical efficacy and if the course would prepare students similarly to previous clinical experience.

Methods

First-year medical students completed a 30-question survey consisting of a Likert scale and free-response questions. Questions were based on the Accreditation Council for Graduate Medical Education core competencies: medical knowledge, practice-based learning and improvement, systems-based practice, patient care, professionalism, and interpersonal and communication skills. Questions on the perceived benefit of the emergency medical responder course and previous clinical experiences were also included. Responses were separated based on participation in the emergency medical responder course and prior clinical experience. Two-tail Welch’s t-tests were performed on the data to determine significance.

Results

Of 98 responses: 20.4% (20/98) of participants of the emergency medical responder course had previous clinical experience, 21.5% (21/98) of participants of the course had no clinical experience, 26.5% (26/98) did not participate in the course but had previous clinical experience, and 31.6% (31/98) did not participate in the course nor had previous clinical experience. Students with previous clinical experience reported the emergency medical responder course improved both their patient care skills and performance in courses that emphasized patient interviewing and physical exams. Students with clinical experience had significantly higher medical knowledge (p < 0.1) and professionalism (p < 0.1) Likert scores. Eighty-seven percent of students agreed the course had a positive impact on their patient care skills.

Conclusion

Larger sample size is needed to make stronger conclusions; however, the responses show the emergency medical responder course had a positive subjective impact on students with previous medical experience. Previous clinical experience leads to the most positive subjective reporting of competencies such as medical knowledge and professionalism. Early clinical exposure, such as an emergency medical responder certification course, may improve self-efficacy and patient care skills for medical students with no previous clinical experience.

## Introduction

In the 2019 American Association of Medical Colleges (AAMC) Matriculating Student Questionnaire, over 65% of matriculating medical students indicated at least one year between college graduation and medical school matriculation [1]. The AAMC recognizes strengthening grade point average (GPA), studying for the Medical Colleges Admissions Test (MCAT), and taking time for reflection and rejuvenation as common uses for gap years [2]. However, there is a hidden benefit to taking a gap year. Coetzee and Bester showed the primary value of a gap year is found in the extra time it allows for finalizing career decisions and personal growth gained by the experience [3]. O’Shea showed delaying higher education for the community, social, or volunteer work allowed students to develop personally, civically, morally, and intellectually. These gains potentially help students take advantage of their university experience [4]. An analysis of medical student interviews by Rashid and Kibble revealed gap years bestow adaptability to change and failure, professional identity formation and understanding of team role, understanding of the “real world,” refocusing of goals, resilience and stress management, and reinforcement of motivation [5]. Lower tolerance for ambiguity has been associated with higher stress levels in medical students. Whereas students with a higher tolerance for ambiguity are more likely to express the desire to work in an underserved area [6]. Furthermore, medical students with higher resilience levels have a better quality of life and perception of the educational environment. Developing resilience helps minimize emotional stress and enhances medical training. Therefore, students who develop these qualities during their gap year clinical experience enter medical school with a higher level of self-efficacy than their peers without a gap year [7].

Studies have indicated early clinical exposure (ECE) improves medical students’ academic strength, clinical skills, and communication skills [8]. When integrated into the medical school curriculum, ECE makes an impact on students’ performance and confidence [9]. Furthermore, ECE is most commonly provided in community settings. When this is true, research shows that experience fosters self-awareness and empathetic attitudes towards the ill, boosts students’ confidence, motivates them, gives them satisfaction, and helps them develop a professional identity. This makes biomedical, behavioral, and social sciences more relevant and easier to learn [10,11]. The integration of an emergency medical responder (EMR) certification course into the first-year curriculum may provide ECE and improved self-efficacy for medical students. In 2019, a week-long EMR course was offered to students at the University of Central Florida (UCF) College of Medicine before their matriculation. Forty-one students voluntarily participated in this course.

Similarly, the Hofstra North Shore-LIJ School of Medicine integrated an Emergency Medical Technician (EMT) curriculum before the matriculation of first-year students to produce early legitimate clinical experience and practice clinical skills as team members before matriculation [12]. Their survey was based on a literature search of patient care skills and team collaboration skills traditionally expected of preclinical medical students. The survey was further validated by emergency medicine faculty and residents who were certified EMTs before entering medical school. The survey identified patient care and team-building skills as well as included core competencies. The Accreditation Council for Graduate Medical Education (ACGME) endorses six core competencies to achieve the expected level of a practitioner [13]. These core competencies are medical knowledge, practice-based learning, improvement, systems-based practice, patient care, professionalism, and interpersonal and communication skills. The study found the incorporation of EMT training early in medical school provides meaningful clinical experiences and increased the self-reported level of confidence in the performance of patient care.

While studies similar to the one at the Hofstra North Shore-LIJ School of Medicine have confirmed the benefits of ECE, none have compared the outcomes of those students with clinical exposure early in their medical curriculum to students with clinical exposure before medical school [12]. The primary objective of this study was to determine the self-reported level of competency and clinical efficacy of students who participated in the EMR course at the UCF College of Medicine. Secondary objectives included comparing these outcomes to those of students with prior clinical experience and determining how self-efficacy plays a role in these outcomes. ECE was defined as exposure during the medical school curriculum as opposed to before the medical school curriculum.

## Materials and methods

This was a cross-sectional study approved by the University of Central Florida College of Medicine Institutional Review Board. Internet surveys were administered to 120 first-year students at the UCF College of Medicine from January 2020 to March 2020 to collect quantitative and qualitative data. Inclusion criteria included first-year medical students at the UCF College of Medicine who were at least 18 years of age at the time of survey administration and were willing to participate. All students could participate in the survey, so outcomes of students who did not complete the EMR certification course could be compared to students who did complete the EMR certification course.

Table 1 displays how questions asked in the survey pertain to the ACGME’s six core competencies. Likert-type scale questions corresponded to numbers for ease of data analysis (1 = strongly disagree, 2 = disagree, 3 = neither agree nor disagree, 4 = agree, and 5 = strongly agree). These survey questions were based on a previous assessment of a month-long emergency medical technician (EMT) course implemented at the Hofstra North Shore LIJ School of Medicine at Hofstra University [12].

**Table 1:** ACGME competency survey questions ACGME: Accreditation Council for Graduate Medical Education

Question	ACGME Competency	Survey Question
1	Medical Knowledge (MK)	I feel prepared for my patient encounters in medical school.
2		I feel confident during my patient encounters in medical school.
3		I feel more prepared than my peers during my patient encounters in medical school.
4	Practice-Based Learning and Improvement (PBLI)	I feel like I perform well in my patient encounters in medical school.
5	Systems-Based Practice (SBP)	I feel comfortable during my patient encounters in medical school.
6	Patient Care (PC)	I am respectful towards patients during my patient encounters in medical school.
7	Professionalism (P)	I attentively listen to patients during my patient encounters in medical school.
8	Interpersonal and Communication Skills (ICS)	I am concise with patients during my patient encounters in medical school.
9		I use non-technical language during my patient encounters in medical school.
10		I communicate effectively during my patient encounters in medical school.
11	Professionalism (P)	I feel accountable during my patient encounters in medical school.
12	Practice-Based Learning and Improvement (PBLI)	I can identify where I need to improve during my patient encounters in medical school.

The survey also assessed how students feel their studies were supplemented by what they learned in the EMR course. Participants had the opportunity to explain why they participated in the course and if they feel the course aided in their patient care skills. Follow-up questions assessed their success during patient encounters. Eleven questions were asked to identify those survey participants who participated in the EMR course, with follow-up questions covering the course’s impact on medical student development, patient care skills, and medical curriculum performance. Five questions were asked to identify those survey participants with previous clinical experience with the experience’s impact on medical student development and patient care skills. Two questions were asked to identify what extracurricular clinical activities survey participants were involved with.

Two-tail Welch’s t-tests were performed on the data to determine significance. Tests for statistical significance were performed and analyzed using IBM Corp. Released 2017. IBM SPSS Statistics for Windows, Version 25.0. Armonk, NY: IBM Corp. For a significant parameter for these tests, a p-value of 0.05 was used.

Quantitative data was collected to quantify the populations who participate in the study. This data indicated who participates in extracurricular clinical activities and the EMR course. Free responses were requested to understand why students participated in the EMR course and how they felt the course affected their patient care skills.

## Results

The total response rate of the survey was 82% (98/120). The total number of EMR participants who completed the survey was 100% (41/41). Of 98 responses: 20 participants of the EMR course had previous clinical experience, 21 participants of the EMR course had no clinical experience, 26 only had previous clinical experience, and 31 did not participate in the course nor had previous clinical experience.

**Figure 1:**
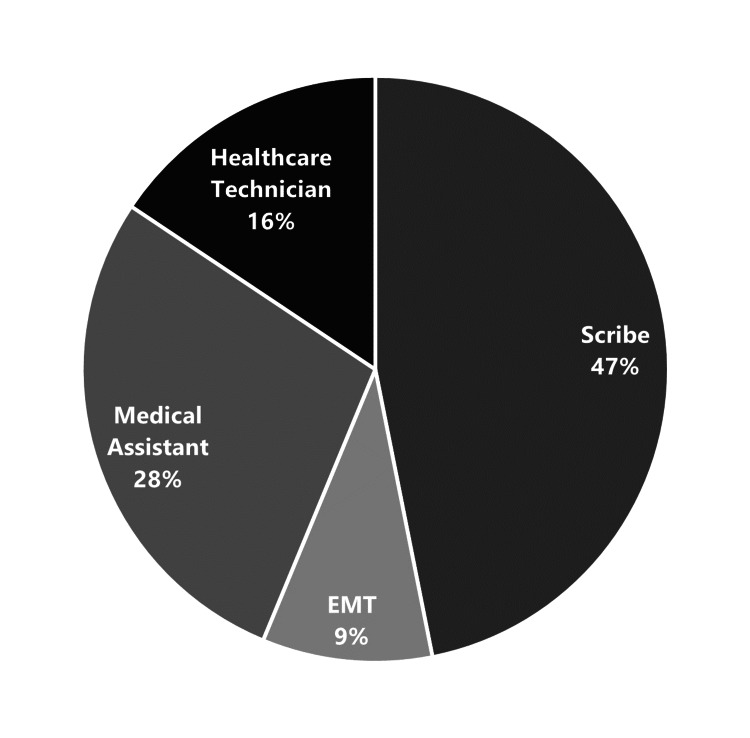
Prior medical experience EMT: Emergency Medical Technician

Forty-five of the 98 respondents (46%) had previous clinical experience, the type of experience is shown in Figure 1. Approximately 97% of responders strongly agreed or agreed their previous clinical experience had been important to their development as medical students.

Students who had clinical experience before beginning medical school in the matriculating class of 2019 reported the EMR course did improve both their patient care skills and performance in the practice of medicine module. There were significant differences in medical knowledge (p < 0.01) and professionalism (p < 0.01) between those with prior clinical experience and those who took the EMR course. There was a significant difference in interpersonal and communication skills (p = 0.04) between the students with only prior clinical experience and students who did not participate in the EMR course nor had previous clinical experience. There were no significant differences in systems-based practice, practice-based learning, improvement, or patient care between any of the groups. Eighty-seven percent of EMR course participants agreed that the EMR course had a positive impact on their patient care skills.

Three themes were identified through analysis of the free-response data of EMR course participants on the benefits of the course: 1) An appreciation for hands-on experiences early in their medical education, 2) Increased confidence in their medical curriculum, and 3) Increased motivation for their medical careers.

Specific free responses to why these students chose to participate in the EMR course and their perceived benefits of the course are listed in Appendix A.

## Discussion

Development of core competencies

Models and simulations are commonplace in an EMR certification course. According to the Council of Emergency Medicine Residency Directors (CORD-EM) outline, these are suggested as an instruction to meet the patient care, medical knowledge, and interpersonal and communication skills competencies [14].

Students with previous clinical experience reported the EMR course improved both their patient care skills and performance in courses that emphasized patient interviewing and physical exams, much like previous studies have found emergency medical services courses improve self-perceived competency [12,15]. Students with clinical experience had significantly higher self-perceived medical knowledge and professionalism scores. A study emphasizing professionalism showed clinical groups of students score higher than preclinical students [16]. The benefits of professionalism are fostering patient adherence and the creation of a healthy working environment [17]. Previous clinical experience not only improved the fund of knowledge for medical students but also provided professional development.

Students who had clinical work before beginning medical school reported the EMR course improved their patient care skills and performance in the practice of medicine module. The group of students who solely became EMR certified had higher Likert scores than the group of students with prior clinical experience who became EMR certified in medical knowledge and patient care, possibly due to a falsely higher perception of their ability due to lack of real-world clinical experience, but this would need to be investigated further. Similarly, a survey of students who completed an EMT course before medical school indicated a significant increase in students' confidence in patient care [18]. “I use non-technical language during my patient encounters in medical school” and “I can identify where I need to improve during my patient encounters in medical school” in the group of students with prior clinical experience who became EMR certified were higher than the group of students with prior clinical experience who did not become EMR certified, indicating increased ability to use appropriate language and identify areas of improvement by EMR participants. There was no significant difference in clinical activity engagement among the groups during their first year. This is somewhat unexpected, given the EMR participants were motivated to begin clinical training before the start of medical school. A possible explanation could be the EMR participants fulfilled their need for early clinical experience in the medical curriculum and did not further seek out clinical activities afterward. 

Development of self-efficacy

A gap year has been proposed as an effective way for students to gain self-efficacy and life skills before starting medical school. A study suggested encouraging participation and strengthening self-efficacy may help enhance medical student performance [19]. While medical education has increased awareness that self-efficacy plays an important role in student learning and development, there is a lack of congruency with conceptual guidelines proposed by self-efficacy experts [20]. Many techniques have been suggested for helping students develop self-efficacy. Suggested institutional practices include 1) encouraging students to set clear, specific, challenging proximal goals, 2) providing students with honest and explicit feedback, 3) facilitating accurate calibration of self-efficacy, and 4) using peer modeling [21]. The results of the survey show increased confidence and motivation of participants in the EMR certification course. An EMR certification course may be able to pass on the benefits of a gap year clinical experience to a first-year medical student. This is especially possible when setting clear goals within the course and giving explicit feedback.

Development of clinical skills

Medical schools such as the University of South Carolina School of Medicine Greenville have integrated an Emergency Medical Technician (EMT) course into their curriculum with generally positive results [22,23]. The implementation allows students to better understand patient populations and positively influence medical education [24]. The main purposes for integration of an EMT curriculum at the Zucker School of Medicine were to 1) provide a foundation of a spiral learning approach, 2) contextualize the basic sciences within clinical practice, 3) provide opportunities to engage in authentic clinical activities under the guidance of mentors, 4) introduce students to the interdisciplinary nature of medicine, and 5) serve as the first entrustable professional activity for students [22].

The EMR certification course showed similar outcomes. Student survey responses supported the early hands-on approach and application of sciences to clinical practice. Likewise, a study of students completing an EMT course before medical school emphasized themes of retention and transferability of practical skills, a developed understanding of team communication, comfort with patient interactions, and the development of a framework for assessing patients' needs [18]. Furthermore, the course provided opportunities to volunteer in the local community and build relationships with emergency medicine health professionals. Additionally, the course motivated and built confidence in the medical curriculum. The EMR course was shown to meet similar goals and purposes as previously implemented EMT courses.

Limitations and future directions

There are several limitations to cross-sectional studies like this one. Since the survey asks about previous experience and student performance at the same time, they are subject to temporal relationship problems. This is problematic for making inferences regarding class-to-performance causality. Cross-sectional studies can be subject to confounding, which can distort the association between exposure of interest (experience) and the outcome (performance). Confounding variables could include the age of participants, as age is associated with increased maturity and confidence.

With the small sample sizes of both the EMR course study participants (41) and total study participants (98), more participants would be beneficial to increase the power of the study. Survey responses are a subjective reflection of how students felt during their medical courses and patient interactions. Future studies should include objective measurements such as final course grades or patient satisfaction ratings to determine if the EMR course is beneficial. Age and its relation to life experience should also be accounted for in future studies to understand the effect on clinical confidence.

## Conclusions

Early clinical experience is an essential part of preclinical medical education because of its contribution to increasing student confidence, motivation, and hands-on skills. Clinical experience before starting medical school significantly increases self-reported competencies such as medical knowledge and professionalism. An EMR certification course can provide similar benefits of self-efficacy and patient care as clinical experience before the first year of medical school. 

## Appendices

Appendix A

Free-response answers to "Please elaborate on how you think the Emergency Medical Responder (EMR) course has affected your patient encounters:"

“The EMR course was my first encounter with medical interviews.”

“It taught practical skills, as opposed to theoretical knowledge, which helped immensely during volunteering as a medical student.”

“It ends up being more important to know how to place a splint than it is to know the mechanism of the area cycle. I think medical school should encourage and prioritize practical skills early.”

“The EMR course refreshed many of my history-taking skills and I was able to apply this to the hurricane shelter.”

“I believe that the EMR course made me feel more confident about the way I conduct patient interviews and provided me with unique hands-on patient encounters during my first year. I think that the EMR course has given me an advantage over my peers in terms of my interviewing and patient rapport skills.”

“I believe that by learning hands-on medical techniques and knowledge about real-world use I had more motivation and a better understanding of how my medical knowledge would be useful.”

“It has helped me to be more comfortable with my hands-on skills, especially in the case of physical examination.”
